# Comparative Evaluation of the Antioxidant and Anti-Inflammatory Activities of *Sargassum horneri* Collected from Different Korean Coastal Regions

**DOI:** 10.3390/md24100339

**Published:** 2026-09-27

**Authors:** Sung-Gyu Lee, Jae-Ho Lee, Hyun Kang

**Affiliations:** 1 Department of Medical Laboratory Science, College of Health Science, Dankook University, Cheonan-si 31116, Chungcheongnam-do, Republic of Korea; sung-gyu@dankook.ac.kr (S.-G.L.); dlwogh1214@naver.com (J.-H.L.); 2 Marine Bio-Food and Drug Convergence Technology Center, Dankook University, Cheonan-si 31116, Chungcheongnam-do, Republic of Korea

**Keywords:** *Sargassum horneri*, geographical variation, antioxidant activity, anti-inflammatory activity, nitric oxide, MAPK, NF-κB

## Abstract

*Sargassum horneri* is a brown alga with diverse biological activities; however, the influence of geographical origin on its functional properties remains poorly understood. This study comparatively evaluated the antioxidant and anti-inflammatory activities of *S. horneri* extracts originating from the East, South, and West Seas of Korea. Total phenolic content (TPC) and total flavonoid content (TFC) were determined, and antioxidant activity was assessed using 2,2-diphenyl-1-picrylhydrazyl (DPPH) and 2,2′-azino-bis(3-ethylbenzothiazoline-6-sulfonic acid) (ABTS) radical-scavenging assays and the ferric reducing antioxidant power (FRAP) assay. Anti-inflammatory activity was evaluated in lipopolysaccharide (LPS)-stimulated RAW 264.7 macrophages. The South Sea extract exhibited the highest TPC and showed strong overall antioxidant activity. LPS stimulation reduced cell viability and markedly increased nitric oxide (NO) production, whereas treatment with the regional extracts improved cell viability and suppressed NO production to varying degrees. Among the three extracts, the South Sea extract exhibited the most pronounced inhibitory effect on LPS-induced NO production. Based on its overall bioactivity profile, the South Sea extract was selected for further mechanistic investigation. The extract reduced cyclooxygenase-2 (COX-2) expression and nuclear factor-kappa B (NF-κB) phosphorylation. Furthermore, extracellular signal-regulated kinase (ERK) phosphorylation was markedly reduced, particularly at 400 μg/mL, while c-Jun N-terminal kinase (JNK) phosphorylation showed an overall decreasing tendency at higher concentrations without a strictly concentration-dependent response. In contrast, p38 phosphorylation was not suppressed. These findings suggest that the anti-inflammatory activity of the South Sea *S. horneri* extract may be associated, at least in part, with modulation of ERK/JNK and NF-κB signaling. Overall, this study demonstrates regional variation in the antioxidant and anti-inflammatory properties of *S. horneri* and identifies the South Sea-derived extract as a promising marine resource for the development of functional materials.

## 1. Introduction

Marine macroalgae are increasingly recognized as valuable sources of structurally diverse bioactive compounds with potential applications in functional foods, nutraceuticals, pharmaceuticals, and cosmeceuticals [1,2,3,4]. Among marine macroalgae, brown algae (Phaeophyceae) are particularly rich in biologically active constituents, including polyphenols, phlorotannins, carotenoids, sterols, and sulfated polysaccharides, many of which exhibit antioxidant, anti-inflammatory, metabolic, and other health-promoting properties [2,3,4,5]. Phenolic compounds derived from brown algae have received considerable attention because of their strong radical-scavenging and reducing activities as well as their potential to modulate cellular processes associated with oxidative stress and inflammation [5,6,7,8,9]. These characteristics have stimulated increasing interest in the identification and utilization of brown algal resources as natural sources of functional ingredients.

*Sargassum horneri* (Turner) C. Agardh is an annual brown alga widely distributed in the coastal waters of East Asia, including Korea, Japan, and China. In recent years, the extensive proliferation and accumulation of *S. horneri*, often referred to as “golden tides,” have raised ecological and socioeconomic concerns, while its abundant biomass has also attracted attention as a potentially valuable marine resource [10,11]. *S. horneri* contains various bioactive constituents, including phenolic compounds, phytosterols, and sulfated polysaccharides, which contribute to its diverse biological properties [12,13,14,15]. In particular, *S. horneri* extracts have demonstrated antioxidant and anti-inflammatory activities, including radical-scavenging effects and suppression of inflammatory mediators in activated macrophages [12,16,17]. Recent studies have further demonstrated that *S. horneri* extracts and their bioactive constituents can modulate oxidative stress- and inflammation-related signaling pathways, including nuclear factor-kappa B (NF-κB), mitogen-activated protein kinases (MAPKs), and nuclear factor erythroid 2-related factor 2/heme oxygenase-1 (Nrf2/HO-1) signaling [13,16,17]. These findings suggest that *S. horneri* is a promising marine resource for the development of functional materials.

Oxidative stress and inflammation are closely interconnected biological processes involved in the development and progression of numerous chronic disorders. Excessive production of reactive oxygen species (ROS) can disrupt cellular redox homeostasis and promote inflammatory responses, whereas sustained inflammation can further exacerbate oxidative stress [6,9]. In macrophages, lipopolysaccharide (LPS) stimulation activates major inflammatory signaling pathways, including NF-κB and MAPKs, resulting in increased expression of inflammatory mediators such as inducible nitric oxide synthase (iNOS) and cyclooxygenase-2 (COX-2) and consequently enhanced production of nitric oxide (NO) and pro-inflammatory cytokines [12,13,18]. Previous studies have demonstrated that *S. horneri* extracts and their bioactive constituents can attenuate inflammatory responses by suppressing inflammatory mediator production and modulating NF-κB and MAPK signaling pathways [12,13,14,18,19,20,21]. These findings further support the potential of *S. horneri* as a source of natural anti-inflammatory agents.

The biochemical composition and biological activities of marine algae, however, are not necessarily constant. Environmental and geographical factors, including seawater temperature, light exposure, nutrient availability, salinity, and other habitat conditions, can influence the biosynthesis and accumulation of secondary metabolites, thereby affecting the functional properties of algal biomass [22,23]. Seasonal variations in phenolic content, antioxidant activity, and other bioactive constituents have been demonstrated in several brown algal species, including members of the genus Sargassum [22,23,24,25]. In particular, temporal variations in total phenolic content and antioxidant capacity have been reported among Sargassum species, suggesting that harvesting period and environmental conditions can substantially influence their functional properties [24]. Moreover, spatial and seasonal differences in the accumulation of biofunctional compounds, including fucoxanthin and fucosterol, have been demonstrated in *S. horneri*, with variations associated with environmental conditions such as temperature and light availability [26]. Despite growing interest in the biological activities of *S. horneri*, comparative information regarding the antioxidant and anti-inflammatory properties of *S. horneri* originating from different Korean coastal regions remains limited. Therefore, understanding geographical variability in the bioactivity of *S. horneri* is important for evaluating the functional quality of its biomass and its potential utilization as a source of bioactive marine materials.

Therefore, the present study comparatively evaluated the antioxidant and anti-inflammatory activities of *S. horneri* extracts originating from different coastal regions of Korea. Total phenolic content (TPC), total flavonoid content (TFC), antioxidant capacity, and NO production were evaluated to compare the bioactivities of the regional extracts. The extract exhibiting the highest overall bioactivity was further investigated for its effects on COX-2 expression and NF-κB and MAPK signaling pathways. These findings provide a basis for understanding regional variability in the functional properties of *S. horneri* and identifying promising resources for further utilization.

## 2. Results

### 2.1. Regional Variation in TPC and TFC of S. horneri Extracts

The TPC and TFC of *S. horneri* extracts originating from three different Korean coastal regions were compared (Figure 1). The South Sea extract exhibited the highest TPC, reaching approximately 87.36 ± 1.38 mg gallic acid equivalents (GAE)/g extract, which was significantly higher than those of the West and East Sea extracts (approximately 36.09 ± 2.60 and 39.86 ± 4.51 mg GAE/g extract, respectively; *p* < 0.05) (Figure 1B). No significant difference in TPC was observed between the West and East Sea extracts.

In contrast, TFC showed a distinct regional pattern (Figure 1C). The East Sea extract exhibited the highest TFC at approximately 23.11 ± 3.77 mg quercetin equivalents (QE)/g extract, followed by the West Sea extract at approximately 11.78 ± 0.38 mg QE/g extract, whereas the South Sea extract showed the lowest value at approximately 8.67 ± 0.58 mg QE/g extract. Significant differences in TFC were observed among all three regional extracts (*p* < 0.05). Overall, these results demonstrate clear regional variation in the phenolic and flavonoid contents of *S. horneri* extracts, with the South Sea extract characterized by the highest TPC and the East Sea extract by the highest TFC.

### 2.2. Regional Variation in Antioxidant Activities of S. horneri Extracts

The antioxidant activities of *S. horneri* extracts originating from different Korean coastal regions were evaluated using 2,2-Diphenyl-1-picrylhydrazyl (DPPH) and 2,2′-azino-bis(3-ethylbenzothiazoline-6-sulfonic acid) (ABTS) radical-scavenging assays and the ferric reducing antioxidant power (FRAP) assay (Figure 2). In the DPPH assay, the South Sea extract exhibited the lowest half-maximal inhibitory concentration (IC_50_) value (approximately 152.99 ± 1.68 μg/mL), which was significantly lower than those of the West and East Sea extracts (approximately 352.93 ± 13.65 and 321.38 ± 39.89 μg/mL, respectively; *p* < 0.05) (Figure 2A). No significant difference was observed between the West and East Sea extracts. A similar pattern was observed in the ABTS radical-scavenging assay (Figure 2B). The South Sea extract showed the lowest IC_50_ value (approximately 53.27 ± 2.43 μg/mL), followed by the West Sea extract (approximately 174.89 ± 8.21 μg/mL) and the East Sea extract (approximately 213.11 ± 12.96 μg/mL). Significant differences were observed among all three regional extracts (*p* < 0.05). The FRAP assay showed that the South and East Sea extracts exhibited significantly higher reducing capacities than the West Sea extract (*p* < 0.05), whereas no significant difference was observed between the South and East Sea extracts (Figure 2C). Collectively, the South Sea extract exhibited the strongest DPPH and ABTS radical-scavenging activities and a high ferric reducing capacity among the regional extracts.

### 2.3. Effects of Regional S. horneri Extracts on Cell Viability and NO Production

The effects of *S. horneri* extracts from different Korean coastal regions on cell viability and NO production were evaluated in LPS-stimulated RAW 264.7 macrophages (Figure 3). LPS stimulation markedly reduced cell viability to 63.34 ± 0.19% compared with the untreated control (Figure 3A). Treatment with all regional *S. horneri* extracts increased cell viability compared with the LPS-treated group, although the extent of recovery differed among the extracts and concentrations. The West Sea extract increased cell viability to 85.32 ± 4.55–88.16 ± 0.16% at 100–400 μg/mL. The South Sea extract showed cell viability of 97.94 ± 2.05%, 93.10 ± 0.66%, and 78.37 ± 7.44% at 100, 200, and 400 μg/mL, respectively. Similarly, the East Sea extract increased cell viability to 71.72 ± 10.20–74.52 ± 1.80% across the tested concentrations. These results indicate that all three regional extracts attenuated the LPS-associated reduction in cell viability, with the South Sea extract showing the greatest recovery, particularly at 100 and 200 μg/mL.

LPS stimulation markedly increased NO production from 6.41 ± 0.17 μM in the untreated control to 18.02 ± 1.27 μM (Figure 3B). Treatment with all three regional *S. horneri* extracts reduced LPS-induced NO production, although the magnitude of the inhibitory effect varied among the extracts. The West Sea extract reduced NO production to 12.97 ± 0.39, 10.91 ± 0.64, and 8.25 ± 0.49 μM at 100, 200, and 400 μg/mL, respectively, showing a concentration-dependent reduction. Notably, the South Sea extract exhibited lower NO levels than the other regional extracts at all tested concentrations, with NO production decreasing to 9.97 ± 0.69, 9.26 ± 0.70, and 7.68 ± 0.22 μM at 100, 200, and 400 μg/mL, respectively. In contrast, the East Sea extract showed relatively modest changes, with NO levels of 10.12 ± 2.49, 10.83 ± 0.70, and 9.97 ± 0.28 μM, respectively. Overall, the South Sea extract exhibited the most pronounced suppression of LPS-induced NO production, particularly at 400 μg/mL.

### 2.4. Integrated Comparison of Regional Bioactivity Profiles

To comprehensively compare the functional properties of *S. horneri* extracts from different Korean coastal regions, the phenolic contents, antioxidant activities, anti-inflammatory activities, and cell viability were integrated using heatmap visualization and hierarchical clustering (Figure 4). Distinct bioactivity profiles were observed among the three regional extracts. The South Sea extract showed high normalized scores for TPC and FRAP and consistently high antioxidant scores after inverse transformation of the DPPH and ABTS IC_50_ values. It also exhibited high NO inhibitory activity across the tested concentrations. In contrast, the East Sea extract was characterized by the highest TFC score and relatively high FRAP activity but showed lower integrated scores for DPPH and ABTS radical-scavenging activities and NO inhibition. The West Sea extract generally displayed intermediate or lower scores across these bioactivity parameters. Hierarchical clustering further demonstrated differences in the overall profiles of the regional extracts. The West and East Sea extracts clustered more closely with each other, whereas the South Sea extract formed a distinct branch. Overall, the integrated analysis identified the South Sea extract as having the most prominent combined antioxidant and anti-inflammatory activity profile among the three regional extracts.

### 2.5. Effect of the South Sea S. horneri Extract on COX-2 Expression

Based on the integrated comparison of antioxidant and anti-inflammatory activities, including TPC, DPPH and ABTS radical-scavenging activities, FRAP reducing capacity, and inhibition of LPS-induced NO production, the South Sea *S. horneri* extract was selected for further mechanistic investigation because it exhibited the most prominent combined bioactivity profile among the three extracts. LPS stimulation markedly increased COX-2 protein expression compared with the untreated control (*p* < 0.0001) (Figure 5). Treatment with the South Sea extract differentially affected COX-2 expression depending on the concentration. At 100 μg/mL, COX-2 expression was significantly increased compared with the LPS-treated group (*p* < 0.0001). In contrast, treatment with 200 μg/mL reduced COX-2 expression to a level comparable to that of the LPS-treated group, with no statistically significant difference. Notably, treatment with 400 μg/mL markedly decreased COX-2 expression compared with the LPS-treated group (*p* < 0.0001), approaching the level observed in the untreated control. These results indicate that the South Sea *S. horneri* extract effectively suppressed LPS-induced COX-2 expression at the highest tested concentration, although the response was not concentration-dependent across the entire concentration range.

### 2.6. Effect of the South Sea S. horneri Extract on NF-κB Phosphorylation

The effect of the South Sea *S. horneri* extract on NF-κB phosphorylation was examined in whole-cell lysates of LPS-stimulated RAW 264.7 macrophages (Figure 6). Western blot analysis showed that total NF-κB protein levels remained relatively stable, whereas the phosphorylation level of NF-κB varied following treatment with the extract (Figure 6A). Quantitative analysis of the Phosphorylated NF-κB (p-NF-κB)/NF-κB ratio revealed no significant difference between the untreated control and LPS-treated groups (Figure 6B). Treatment with 100 μg/mL of the South Sea extract significantly reduced the p-NF-κB/NF-κB ratio compared with the LPS-treated group, whereas treatment with 200 μg/mL did not show a similar suppressive effect. At 400 μg/mL, the extract markedly reduced the p-NF-κB/NF-κB ratio compared with the LPS-treated group. These findings indicate that the South Sea *S. horneri* extract modulated NF-κB phosphorylation, with the strongest inhibitory effect observed at 400 μg/mL.

### 2.7. Effect of the South Sea S. horneri Extract on MAPK Phosphorylation

To further investigate the signaling mechanisms associated with the anti-inflammatory activity of the South Sea *S. horneri* extract, the phosphorylation of MAPKs, including extracellular signal-regulated kinase (ERK), c-Jun N-terminal kinase (JNK), and p38, was examined in LPS-stimulated RAW 264.7 macrophages (Figure 7). LPS stimulation markedly increased ERK phosphorylation compared with the untreated control. Treatment with the South Sea extract reduced ERK phosphorylation, with the most pronounced reduction observed at 400 μg/mL, while total ERK expression remained relatively unchanged. JNK phosphorylation also showed an overall decreasing tendency at higher extract concentrations, with a marked reduction observed at 200 μg/mL; however, this response was not strictly concentration-dependent. In contrast, p38 phosphorylation was not suppressed and increased at 200 and 400 μg/mL. Notably, the suppression of ERK phosphorylation, together with the reduction in JNK phosphorylation, was accompanied by decreased NF-κB phosphorylation (Figure 6). These findings suggest that the anti-inflammatory activity of the South Sea *S. horneri* extract may be associated primarily with modulation of ERK and JNK signaling and subsequent regulation of NF-κB activation, rather than uniform suppression of all MAPK pathways.

## 3. Discussion

The present study demonstrated that *S. horneri* extracts originating from different Korean coastal regions exhibit distinct phenolic profiles and antioxidant and anti-inflammatory activities. Among the three regional extracts, the South Sea extract showed the highest TPC, strong radical-scavenging and reducing capacities, and the most pronounced suppression of LPS-induced NO production. Integrated heatmap and hierarchical clustering analyses further distinguished the South Sea extract from the West and East Sea extracts based on its overall bioactivity profile. These findings demonstrate differences in the functional properties among *S. horneri* resources obtained from different Korean coastal regions; however, these differences cannot be attributed solely to geographical origin because the samples were collected at different times and in different years. Environmental factors such as temperature, light, salinity, nutrients, hydrodynamic conditions, and seasonality can influence the accumulation of primary and secondary metabolites in brown algae [22,27]. In the present study, the West Sea sample was collected in June 2021, whereas the South and East Sea samples were collected in April 2022 and April 2023, respectively. Therefore, geographical origin and collection timing were not independently controlled, and potential seasonal and interannual effects on the observed phenolic profiles and biological activities cannot be excluded. Accordingly, the present findings should be interpreted as a comparative evaluation of the three regional resources examined rather than as definitive evidence of geographical effects alone. A recent study of *Sargassum thunbergii* collected from different Korean coastal regions similarly demonstrated substantial regional variation in phenolic content, antioxidant capacity, and anti-inflammatory activity [27].

A notable feature of the present results was the difference between TPC and TFC among the regional extracts. The South Sea extract exhibited the highest TPC, whereas the East Sea extract showed the highest TFC (Figure 1). Nevertheless, the South Sea extract displayed stronger overall antioxidant activity, particularly in the DPPH and ABTS radical-scavenging assays. Notably, the FRAP assay showed a somewhat different regional pattern, with the South and East Sea extracts exhibiting comparable reducing capacities, both of which were significantly higher than that of the West Sea extract (Figure 2). This difference among antioxidant assays may reflect the distinct reaction mechanisms assessed by each method; DPPH and ABTS primarily evaluate radical-scavenging activity, whereas FRAP reflects the electron-donating and reducing capacity of the extracts. Thus, the antioxidant profile of the South Sea extract was not uniformly superior across all assays, although it showed the strongest DPPH and ABTS radical-scavenging activities. Brown algae contain a broad range of antioxidant metabolites, including polyphenols, phlorotannins, carotenoids, meroterpenoids, phytosterols, and polysaccharides [5,6,7,8,9,28,29]. Among these compounds, phlorotannins are particularly important antioxidant constituents of brown algae because their phenolic hydroxyl groups contribute to radical-scavenging and reducing activities [6,7,8,29]. Therefore, the higher TPC of the South Sea extract may be associated with its strong antioxidant activity; however, because only three independent regional resources were examined, a robust correlation analysis between TPC/TFC and individual antioxidant parameters could not be performed. Thus, the present study does not establish a quantitative relationship between phenolic/flavonoid contents and antioxidant capacity.

The antioxidant potential of *S. horneri* observed in the present study is consistent with previous reports [15,16,17]. Javed et al. demonstrated that polyphenol-rich *S. horneri* extracts exhibited potent antioxidant properties associated with MAPK/Nrf2/HO-1 signaling [15], whereas Fan et al. reported antioxidant effects of *S. horneri* fucoidan oligosaccharides [16]. More recently, Shin et al. demonstrated that solvent fractionation of ethanolic *S. horneri* extract substantially altered phenolic and flavonoid contents as well as DPPH and ABTS radical-scavenging activities [30]. This finding further suggests that differences in chemical composition can markedly influence the antioxidant properties of *S. horneri*. Comparable associations between phenolic composition and antioxidant activity have also been reported in other brown seaweeds. In *Fucus distichus* collected from different Arctic seas, substantial geographical variation was observed in biochemical composition and phenolic content, and samples with higher phenolic contents generally exhibited stronger DPPH radical-scavenging activity [31]. Similarly, seasonal and geographical variations in biochemical composition, phenolic content, and antioxidant activity have been reported for *Fucus spiralis*, supporting the influence of environmental and harvesting conditions on the functional properties of brown algae [32]. More recently, Horta et al. demonstrated that the phenolic content and antioxidant activity of *F. spiralis*, as assessed by DPPH and FRAP assays, varied markedly depending on the extraction method and conditions, further highlighting the influence of sample processing on the recovery and antioxidant properties of brown algal phenolic compounds [33].Furthermore, *F. vesiculosus* and *Ascophyllum nodosum*, which are rich in phlorotannins, have demonstrated pronounced DPPH, ABTS, and FRAP antioxidant activities, further supporting the contribution of phenolic constituents to the antioxidant properties of brown seaweeds [34,35]. More recently, *A. nodosum* collected from the Barents, Irminger, and Norwegian Seas was shown to exhibit geographical and reproductive-stage-dependent differences in biochemical composition, including variations in fucoidan-associated carbohydrates, polyphenols, and flavonoids. Notably, free radical-scavenging activity was positively associated with polyphenol content, further supporting the relationship between geographical variability in biochemical composition and the antioxidant potential of brown algae [36]. These observations are consistent with the present finding that the South Sea *S. horneri* extract, which exhibited the highest TPC, also showed the strongest DPPH and ABTS radical-scavenging activities. However, direct quantitative comparisons among species should be made cautiously because antioxidant activity can be influenced by species, geographical and seasonal conditions, extraction procedures, and analytical methods. Importantly, the three extracts used in the present study were prepared under the same extraction conditions, thereby minimizing extraction-related variability and facilitating comparison of the biological properties of resources originating from different coastal regions.

Regional differences were also evident in the cellular assays. LPS stimulation reduced RAW 264.7 cell viability to 63.34% of the untreated control, whereas treatment with all three regional extracts increased cell viability relative to the LPS-treated group. The South Sea extract showed particularly pronounced recovery at 100 and 200 μg/mL. However, cell viability decreased with increasing concentrations of the South Sea extract, reaching 78.37 ± 7.44% at 400 μg/mL, suggesting that some loss of cell viability occurred at the highest concentration. In parallel, LPS stimulation increased NO production from 6.41 to 18.02 μM. All three extracts attenuated LPS-induced NO production, with the South Sea extract reducing NO levels to 9.97, 9.26, and 7.68 μM at 100, 200, and 400 μg/mL, respectively. These findings are consistent with previous studies demonstrating anti-inflammatory effects of *S. horneri* extracts and their bioactive constituents in activated macrophages [12,13,18,19,21]. In particular, *S. horneri*-derived sargachromenol has been reported to suppress macrophage inflammatory responses, further supporting the anti-inflammatory potential of this species [13,18].

The anti-inflammatory activity observed for the South Sea extract was further supported by changes in COX-2 expression. COX-2 is an inducible inflammatory enzyme whose expression increases following inflammatory stimulation and is commonly used as a molecular indicator of macrophage activation. In the present study, COX-2 expression was markedly reduced at 400 μg/mL of the South Sea extract. Notably, COX-2 expression was significantly increased at 100 μg/mL compared with the LPS-treated group, whereas no significant reduction was observed at 200 μg/mL and a marked decrease was observed at 400 μg/mL. This non-monotonic response indicates that the effect of the South Sea extract on COX-2 expression did not follow a simple dose-dependent inhibitory pattern. The increased COX-2 expression at 100 μg/mL may reflect a complex cellular response to the crude extract; however, the underlying mechanism cannot be determined from the present data and requires further investigation. However, because cell viability was also reduced at 400 μg/mL, the pronounced decreases in NO production and COX-2 expression observed at this concentration should be interpreted with caution. Although cell viability remained higher than that of the LPS-treated group, the possibility that reduced cell viability contributed, at least in part, to these inhibitory effects cannot be excluded. Taken together, these findings indicate that the effects on COX-2 should be interpreted cautiously rather than as evidence of uniform anti-inflammatory activity across all tested concentrations. In comparison, fucoidan isolated from *Fucus vesiculosus* has been reported to inhibit both COX-1 and COX-2 activities in a concentration-dependent manner, with IC_50_ values of 27 and 4.3 μg/mL, respectively, indicating greater inhibitory activity toward COX-2 [37]. However, these IC_50_ values were obtained using direct enzyme inhibition assays with isolated fucoidan and therefore cannot be directly compared with the present results obtained from COX-2 protein expression in LPS-stimulated RAW 264.7 macrophages treated with a crude *S. horneri* extract. Nevertheless, these findings support the potential of brown algal constituents to modulate COX-associated inflammatory responses. Similar regulation of inflammatory mediators has been reported for bioactive compounds derived from several Sargassum species. Fucoidan from *S. autumnale* suppressed inflammatory responses in LPS-stimulated RAW 264.7 macrophages through regulation of NF-κB and MAPK signaling [38], while low-molecular-weight fucoidan from *S. siliquastrum* inhibited inflammatory mediator production through related pathways [39]. In addition, sargahydroquinoic acid and sargachromenol from Sargassum yezoense have been shown to contribute to the anti-inflammatory activity of the algal extract [40].

MAPK and NF-κB signaling pathways are important regulators of inflammatory responses in macrophages and are frequently targeted by bioactive compounds derived from brown algae [38,39,40,41,42]. In the present study, the South Sea extract modulated MAPK phosphorylation, with inhibitory effects observed particularly for ERK and JNK. NF-κB phosphorylation was also reduced, with a marked reduction observed at 400 μg/mL. Given the concurrent reduction in cell viability at this concentration, the changes in NF-κB and MAPK phosphorylation at 400 μg/mL cannot be attributed exclusively to specific modulation of inflammatory signaling. These findings are consistent with previous studies demonstrating that brown algal constituents can regulate both MAPK and NF-κB signaling [14,38,39,40,41,42]. For example, fucoidans from *S. autumnale* and *S. siliquastrum* suppressed MAPK/NF-κB signaling in LPS-stimulated macrophages [38,39], while fucoidan from *Padina arborescens* similarly attenuated inflammatory responses in macrophages [41]. More broadly, recent studies support NF-κB and MAPKs as important signaling targets underlying the anti-inflammatory properties of algal bioactive compounds [42]. Comparable anti-inflammatory mechanisms have been reported for *Fucus vesiculosus*. Phlorotannin-rich fractions from *F. vesiculosus* suppressed LPS-induced NO production and the expression of inflammatory mediators, including iNOS and COX-2, in RAW 264.7 macrophages and inhibited IκBα phosphorylation and degradation, thereby preventing NF-κB activation [43]. These findings are consistent with the reductions in NO production, COX-2 expression, and NF-κB phosphorylation observed for the South Sea *S. horneri* extract in the present study. In addition, fucoidan derived from *F. vesiculosus* has demonstrated anti-inflammatory effects in both in vitro macrophage models and an in vivo LPS-induced mouse model, including modulation of pro- and anti-inflammatory mediators and attenuation of macrophage activation [44]. More recently, fucoidans isolated from several brown seaweeds, including *F. vesiculosus*, were shown to inhibit protein denaturation in a concentration-dependent manner [45]. The inhibitory activity was strongly correlated with fucose content and moderately correlated with sulfate content. Notably, purified *F. vesiculosus* fucoidan (FV2) exhibited an IC_50_ of 0.20 mg/mL, compared with 0.37 mg/mL for diclofenac sodium. These findings further demonstrate that the anti-inflammatory potential of brown algal fucoidans may be influenced by their chemical composition and provide additional evidence for the anti-inflammatory properties of *F. vesiculosus*-derived fucoidan. Collectively, these studies indicate that different classes of bioactive constituents from brown algae, particularly phlorotannins and fucoidans, can modulate inflammatory responses through NF-κB- and MAPK-associated pathways.

The concurrent changes in MAPK and NF-κB phosphorylation suggest that modulation of MAPK-associated NF-κB signaling may contribute to the anti-inflammatory effects of the South Sea *S. horneri* extract. However, this interpretation should be made cautiously because specific MAPK inhibitors or gene-silencing approaches were not employed in the present study. Therefore, the present findings demonstrate an association between MAPK and NF-κB modulation rather than establishing a direct causal relationship between these pathways. Collectively, the results suggest that the South Sea *S. horneri* extract exerts anti-inflammatory effects, at least in part, through modulation of MAPK/NF-κB signaling, leading to reduced COX-2 expression and NO production, as summarized in the proposed mechanism (Figure 8).

The regional differences observed in this study may reflect variations in the chemical composition of *S. horneri* biomass. Brown algal metabolite profiles are highly responsive to environmental conditions, and variation in phenolics, polysaccharides, pigments, and other secondary metabolites can consequently influence biological activity [22,27,28,29]. Moreover, the biological activities of brown seaweed polysaccharides are strongly influenced by structural properties such as molecular weight, monosaccharide composition, sulfate content, and branching characteristics [46]. Thus, the superior overall bioactivity of the South Sea extract is likely to reflect the combined contribution of multiple bioactive constituents rather than a single compound.

Several limitations of the present study should be acknowledged. First, chemical characterization was limited to TPC and TFC, and individual bioactive constituents responsible for the regional differences were not identified. Further high-performance liquid chromatography or liquid chromatography–mass spectrometry-based profiling would therefore be valuable for identifying compounds associated with the observed bioactivity of the South Sea extract. Second, only three regional resources were examined, and the samples were collected at different times and in different years. Therefore, geographical origin and collection timing were not independently controlled, and the observed differences cannot be attributed exclusively to geographical origin. In addition, detailed environmental information, including water temperature, salinity, nutrient availability, and light conditions at the time of collection, was not available. Future studies using samples collected during the same period and year across multiple regions will be necessary to more clearly distinguish geographical effects from seasonal and interannual variation. Third, mechanistic experiments were performed only with the selected South Sea extract, and direct causal relationships among MAPK modulation, NF-κB signaling, and downstream inflammatory mediators were not established. In addition, the reduced cell viability observed at 400 μg/mL of the South Sea extract represents a potential confounding factor when interpreting the pronounced anti-inflammatory and signaling effects at this concentration. Finally, the biological activity was evaluated using an in vitro macrophage model; consequently, additional in vivo studies will be required to establish the physiological relevance of these findings. Recent reviews similarly emphasize the importance of detailed chemical characterization, structure–activity analysis, and biological validation when developing brown algal bioactives for functional or pharmaceutical applications [42,46].

Despite these limitations, the present study provides comparative evidence that the antioxidant and anti-inflammatory properties of *S. horneri* vary according to the geographical origin of the algal resource. Among the three Korean coastal regions examined, the South Sea extract exhibited the most favorable overall bioactivity profile, characterized by high phenolic content, strong antioxidant capacity, and pronounced suppression of LPS-induced NO production. The accompanying modulation of COX-2 expression and MAPK/NF-κB signaling further supports its anti-inflammatory potential. These findings highlight the importance of geographical origin in the selection of *S. horneri* resources and provide a basis for further chemical characterization and development of this abundant marine biomass as a source of functional materials.

## 4. Materials and Methods

### 4.1. Preparation of S. horneri Extracts

*S. horneri* extracts collected from three different coastal regions of Korea were obtained from the National Marine Biodiversity Institute of Korea (MABIK, Seocheon, Republic of Korea) (Table 1). The algal materials originated from Taean-gun, Chungcheongnam-do (West Sea; MABIK No. NP30220010), Jeju-si, Jeju-do (South Sea; MABIK No. NP30220007), and Yangyang-gun, Gangwon-do (East Sea; MABIK No. NP30230180). According to the MABIK resource information, the West Sea sample was collected on 10 June 2021, the South Sea sample on 18 April 2022, and the East Sea sample on 19 April 2023. Thus, the three regional samples were collected in different years and were not temporally matched. For all samples, the whole algal thallus was used for extraction. The extracts supplied by MABIK were prepared using 70% ethanol by ultrasonic extraction under standardized extraction conditions. The obtained extracts were stored at −20 °C until further analysis.

### 4.2. Chemicals and Reagents

Folin–Ciocalteu reagent, gallic acid, aluminum chloride, quercetin, DPPH, ABTS, potassium persulfate, 2,4,6-tripyridyl-s-triazine (TPTZ), ferric chloride, Griess reagent, and LPS were purchased from Sigma-Aldrich (St. Louis, MO, USA). Dulbecco’s modified Eagle’s medium (DMEM), fetal bovine serum (FBS), and penicillin–streptomycin were obtained from Gibco (Grand Island, NY, USA). Primary antibodies against COX-2, phosphorylated and total NF-κB, and phosphorylated and total ERK, JNK, and p38 MAPK, as well as β-actin, were obtained from Cell Signaling Technology (Danvers, MA, USA). Horseradish peroxidase (HRP)-conjugated secondary antibodies were purchased from Cell Signaling Technology. All other chemicals and reagents were of analytical grade.

### 4.3. Determination of TPC

The TPC of the *S. horneri* extracts was quantified using the Folin–Ciocalteu colorimetric assay with minor modifications [47]. Each extract was diluted two-fold, and 60 μL of the diluted sample was combined with an equal volume of Folin–Ciocalteu reagent. After incubation for 3 min at room temperature (RT), 60 μL of 10% sodium carbonate (Na_2_CO_3_) solution was added. The mixture was subsequently incubated at RT for 40 min, and the absorbance was recorded at 700 nm using a microplate reader. Gallic acid was used to construct the calibration curve, and the results were expressed as milligrams of gallic acid equivalents per gram of extract (mg GAE/g extract). All measurements were performed in triplicate.

### 4.4. Determination of TFC

The TFC of the *S. horneri* extracts was determined using an aluminum-based colorimetric assay with minor modifications [48]. Briefly, 100 μL of each sample solution was mixed with 20 μL of 10% aluminum nitrate and 20 μL of 1 M potassium acetate. Subsequently, 860 μL of 80% ethanol was added, and the reaction mixture was incubated at RT for 40 min. Absorbance was measured at 415 nm using a microplate reader. Quercetin was used for preparation of the standard calibration curve, and TFC was expressed as milligrams of quercetin equivalents per gram of extract (mg QE/g extract). All measurements were performed in triplicate.

### 4.5. DPPH Radical-Scavenging Activity

The DPPH radical-scavenging activity of the *S. horneri* extracts was evaluated according to the method of Blois [49], with minor modifications. Briefly, 160 μL of each extract at different concentrations was mixed with 40 μL of 0.15 mM DPPH solution. The reaction mixture was incubated at RT for 30 min, after which the absorbance was measured at 517 nm. The radical-scavenging activity was calculated relative to the control prepared without the extract. The antioxidant capacity was expressed as the half-maximal inhibitory concentration (IC_50_), defined as the extract concentration required to scavenge 50% of DPPH radicals.

### 4.6. ABTS Radical-Scavenging Activity

The ABTS radical-scavenging activity was determined according to the method of Re et al. [50], with minor modifications. The ABTS radical cation (ABTS^•+^) solution was generated by mixing 7 mM ABTS with 2.45 mM potassium persulfate and allowing the mixture to react in the dark at RT for 24 h. Before use, the ABTS^•+^ solution was diluted with distilled water to obtain an absorbance of 0.70 ± 0.02 at 732 nm. Subsequently, 10 μL of each extract solution was added to 990 μL of the diluted ABTS^•+^ solution. After incubation at RT for 1 min, the absorbance was recorded at 732 nm. ABTS radical-scavenging activity was calculated relative to the control and expressed as the IC_50_ value, representing the extract concentration required to scavenge 50% of ABTS radicals.

### 4.7. FRAP Assay

The FRAP of the *S. horneri* extracts was determined according to the method of Benzie and Strain [51], with minor modifications. The FRAP working reagent was freshly prepared by combining 300 mM sodium acetate buffer (pH 3.6), 10 mM TPTZ dissolved in 40 mM HCl, and 20 mM FeCl_3_ solution at a ratio of 10:1:1 (*v*/*v*/*v*). For the assay, 10 μL of each extract at the indicated concentration was mixed with 200 μL of freshly prepared FRAP reagent and incubated at 37 °C for 5 min. The absorbance was subsequently measured at 593 nm using a microplate spectrophotometer (xMark™, Bio-Rad, Hercules, CA, USA). A standard calibration curve was prepared using FeSO_4_·H_2_O at concentrations ranging from 0 to 5 mM, and the reducing capacity of each extract was calculated from the standard curve. The results were expressed as FeSO_4_ equivalents (mM/mg extract). All measurements were performed in triplicate.

### 4.8. Cell Culture

RAW 264.7 murine macrophages were obtained from the Korean Cell Line Bank (KCLB, Seoul, Republic of Korea). Cells were maintained in DMEM supplemented with 10% FBS and 1% penicillin–streptomycin. Cultures were maintained at 37 °C in a humidified atmosphere containing 5% CO_2_ and were routinely subcultured upon reaching approximately 70–80% confluence. Cells within 5–6 passages were used for the experiments.

### 4.9. Cell Viability Assay

The effects of the regional *S. horneri* extracts on RAW 264.7 cell viability were assessed using the 3-(4,5-dimethylthiazol-2-yl)-2,5-diphenyltetrazolium bromide (MTT) assay with minor modifications [52]. RAW 264.7 cells were seeded in 96-well plates at a density of 8 × 10^3^ cells/well and allowed to stabilize before treatment. The cells were treated with the West, South, or East Sea *S. horneri* extracts at concentrations of 100, 200, or 400 μg/mL in the presence of LPS (100 ng/mL) for 24 h. Subsequently, 20 μL of MTT solution (5 mg/mL) was added to each well, followed by incubation for 4 h at 37 °C. The resulting formazan crystals were dissolved in dimethyl sulfoxide (DMSO), and absorbance was measured at 570 nm using a microplate reader. Cell viability was calculated relative to the untreated control and expressed as a percentage. The assay was performed in three independent experiments (*n* = 3), and the data are presented as the mean ± SD.

### 4.10. Measurement of NO Production

NO production was assessed by measuring nitrite accumulation in the culture medium using the Griess reaction with minor modifications [53]. RAW 264.7 cells were seeded in 96-well plates and treated with the West, South, or East Sea *S. horneri* extracts at concentrations of 100, 200, or 400 μg/mL, followed by stimulation with LPS (100 ng/mL) for 24 h. After incubation, the culture supernatants were collected and reacted with Griess reagent. Absorbance was measured at 540 nm using a microplate reader, and nitrite concentrations were calculated from a sodium nitrite (NaNO_2_) standard calibration curve. The results were expressed as micromolar (μM) nitrite concentrations. The assay was performed in three independent experiments (*n* = 3), and the data are presented as the mean ± SD.

### 4.11. Western Blot Analysis

RAW 264.7 cells were seeded in 6-well plates at a density of 5 × 10^5^ cells/mL and cultured for 24 h. For COX-2 expression analysis, the cells were treated with the South Sea *S. horneri* extract (100, 200, or 400 μg/mL) in the presence of LPS (100 ng/mL) for 24 h. For analysis of NF-κB and MAPK phosphorylation, including ERK, JNK, and p38 MAPK, cells were treated with the South Sea *S. horneri* extract in the presence of LPS (100 ng/mL) for 30 min. Following treatment, the cells were washed with phosphate-buffered saline (PBS) and lysed in an appropriate lysis buffer. Cell lysates were maintained at 4 °C for 30 min and subsequently centrifuged at 13,000 rpm for 15 min at 4 °C. The resulting supernatants were collected, and protein concentrations were determined prior to electrophoresis. Equal amounts of protein were separated by sodium dodecyl sulfate-polyacrylamide gel electrophoresis (SDS-PAGE) and transferred onto nitrocellulose membranes. The membranes were blocked with 5% skim milk at RT for 1 h and subsequently incubated with the appropriate primary antibodies overnight at 4 °C. After washing with Tris-buffered saline containing Tween-20 (TBST), the membranes were incubated with HRP-conjugated secondary antibodies for 2 h at RT. Protein bands were detected using an enhanced chemiluminescence (ECL) detection system. Protein expression and phosphorylation levels were determined using antibodies against COX-2, phosphorylated and total NF-κB, phosphorylated and total ERK, JNK, and p38 MAPK. β-Actin was used as a loading control. Band intensities were quantified by densitometric analysis, and phosphorylated protein levels were normalized to their corresponding total protein levels. Western blot analyses were performed in three independent experiments (*n* = 3), and the densitometric data represent the mean ± SD of these independent experiments.

### 4.12. Statistical Analysis

All experimental data are presented as the mean ± standard deviation (SD) of three independent experiments. Statistical differences among groups were evaluated using one-way analysis of variance (ANOVA), followed by Duncan’s multiple range test for multiple comparisons. Differences were considered statistically significant at *p* < 0.05. Statistical analyses were performed using SPSS software (version 25.0; IBM Corp., Armonk, NY, USA). For the integrated heatmap and hierarchical clustering analyses, the mean values obtained from the three independent experiments were used. DPPH and ABTS IC_50_ values were inversely transformed so that higher values represented stronger antioxidant activity, and NO data were expressed as inhibition relative to the LPS-stimulated control. All variables were normalized to a scale of 0–1 prior to integration. Hierarchical clustering was performed using Euclidean distance and Ward’s minimum variance method (Ward.D2). Heatmap visualization and hierarchical clustering were performed using R software (version 4.5.2; R Foundation for Statistical Computing, Vienna, Austria) based on the normalized data.

## 5. Conclusions

This study demonstrated regional differences in the antioxidant and anti-inflammatory activities of *S. horneri* originating from Korean coastal waters. Among the three regional resources, the South Sea extract exhibited the most favorable overall bioactivity profile, with high phenolic content, strong antioxidant capacity, and pronounced suppression of lipopolysaccharide-induced nitric oxide production. The South Sea extract also reduced COX-2 expression and modulated MAPK/NF-κB signaling in RAW 264.7 macrophages. These findings highlight geographical origin as an important factor influencing the functional properties of *S. horneri* and suggest that South Sea-derived *S. horneri* may represent a promising marine resource for the development of functional materials. Further chemical characterization and in vivo validation are required to identify the active constituents and confirm their biological relevance.

## Figures and Tables

**Figure 1:**
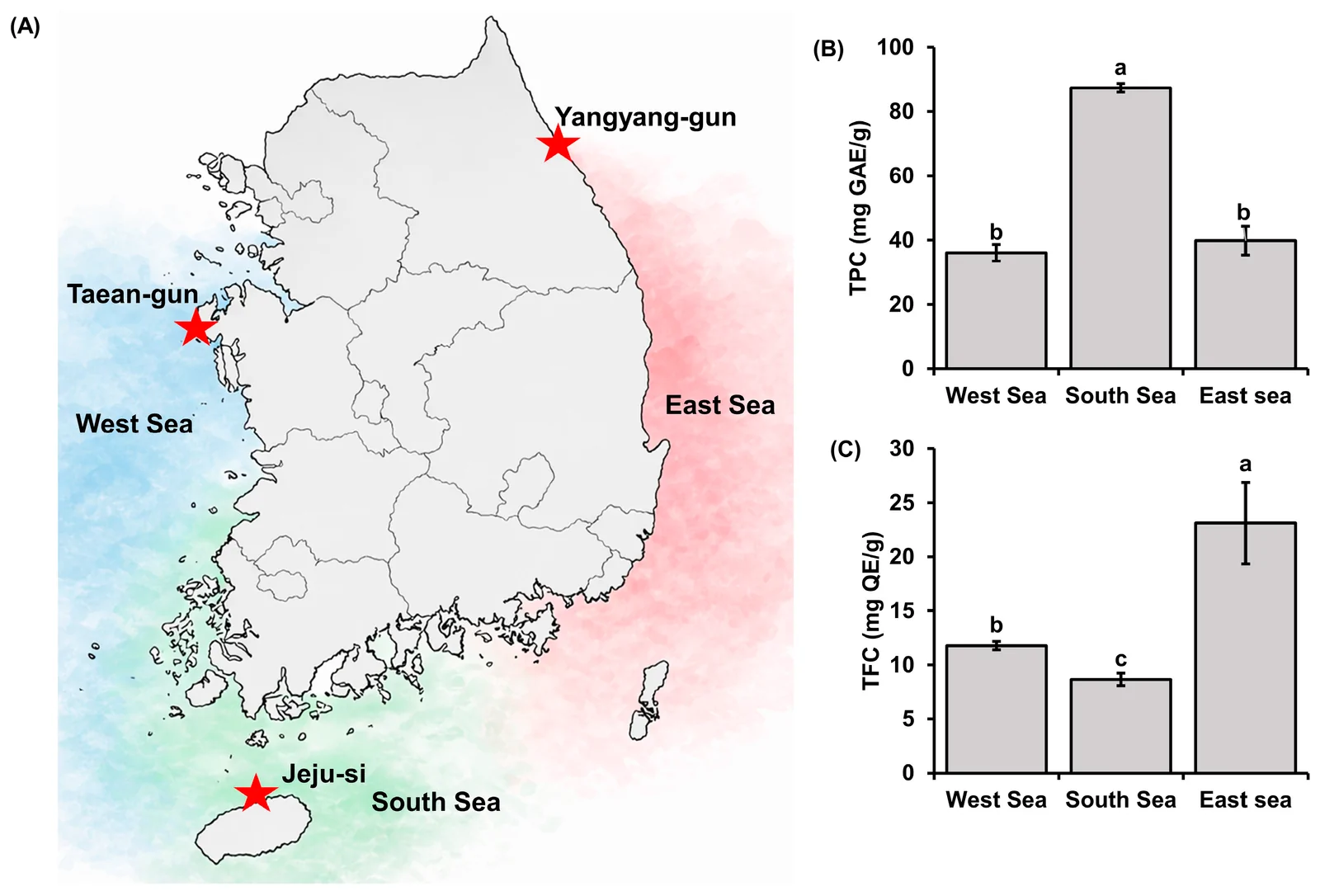
Geographic origins and total phenolic and flavonoid contents of *S. horneri* extracts from different Korean coastal regions. (**A**) Geographic locations of *S. horneri* samples originating from Taean-gun (West Sea), Jeju-si (South Sea), and Yangyang-gun (East Sea), Republic of Korea. Red stars indicate the sampling locations. (**B**) TPC and (**C**) TFC of the regional *S. horneri* extracts. TPC and TFC are expressed as mg GAE/g extract and mg QE/g extract, respectively. Data are presented as the mean ± SD of triplicate measurements. Different lowercase letters indicate statistically significant differences among groups (*p* < 0.05), whereas groups sharing the same letter are not significantly different.

**Figure 2:**
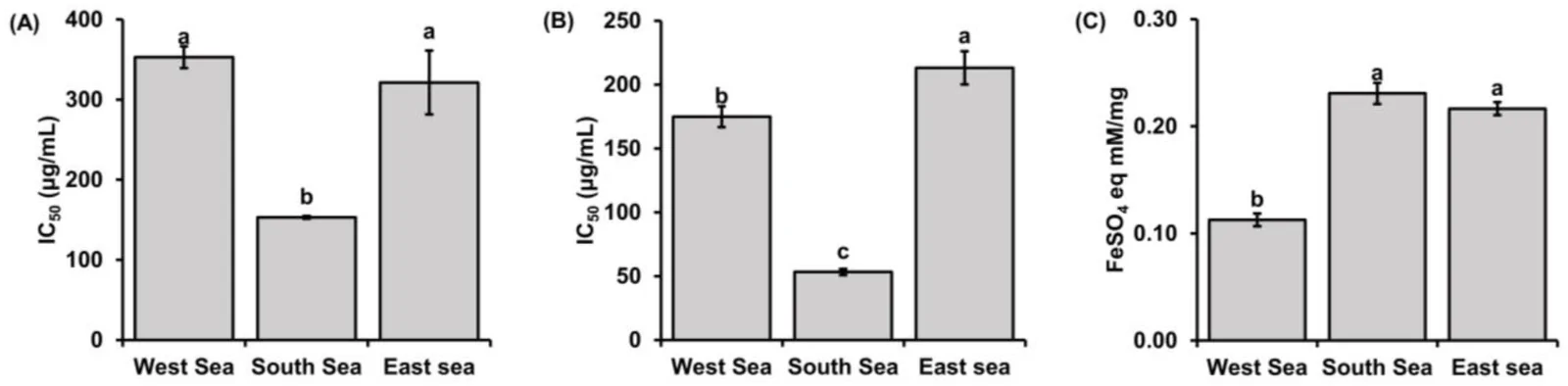
Antioxidant activities of *S. horneri* extracts from different Korean coastal regions. Antioxidant activities of the West, South, and East Sea extracts were evaluated using (**A**) DPPH radical-scavenging, (**B**) ABTS radical-scavenging, and (**C**) FRAP assays. DPPH and ABTS radical-scavenging activities are expressed as IC_50_ values (μg/mL). FRAP values are expressed as FeSO_4_ equivalents (mM/mg extract). Data are presented as the mean ± SD of triplicate measurements. Different lowercase letters indicate statistically significant differences among groups (*p* < 0.05), whereas groups sharing the same letter are not significantly different.

**Figure 3:**
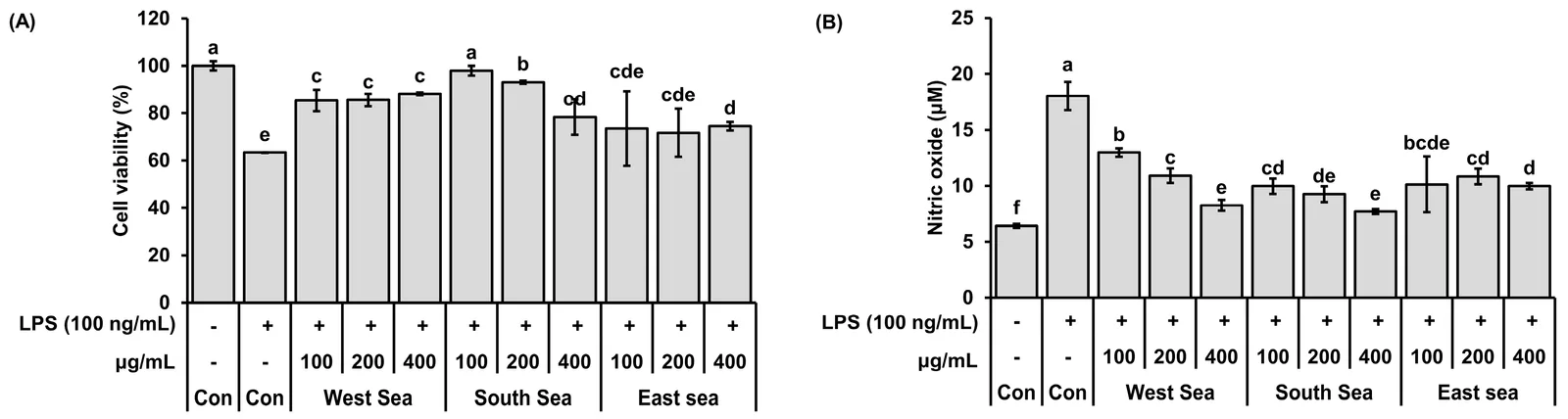
Effects of *S. horneri* extracts from different Korean coastal regions on cell viability and nitric oxide production in LPS-stimulated RAW 264.7 macrophages. RAW 264.7 cells were treated with *S. horneri* extracts from the West, South, and East Seas at concentrations of 100, 200, and 400 μg/mL in the presence of lipopolysaccharide (LPS; 100 ng/mL). (**A**) Cell viability and (**B**) NO production were determined using the MTT assay and Griess reaction, respectively. “+” and “−” indicate the presence and absence of the indicated treatment, respectively. Data are presented as the mean ± SD of triplicate measurements. Different lowercase letters indicate statistically significant differences among groups (*p* < 0.05), whereas groups sharing the same letter are not significantly different.

**Figure 4:**
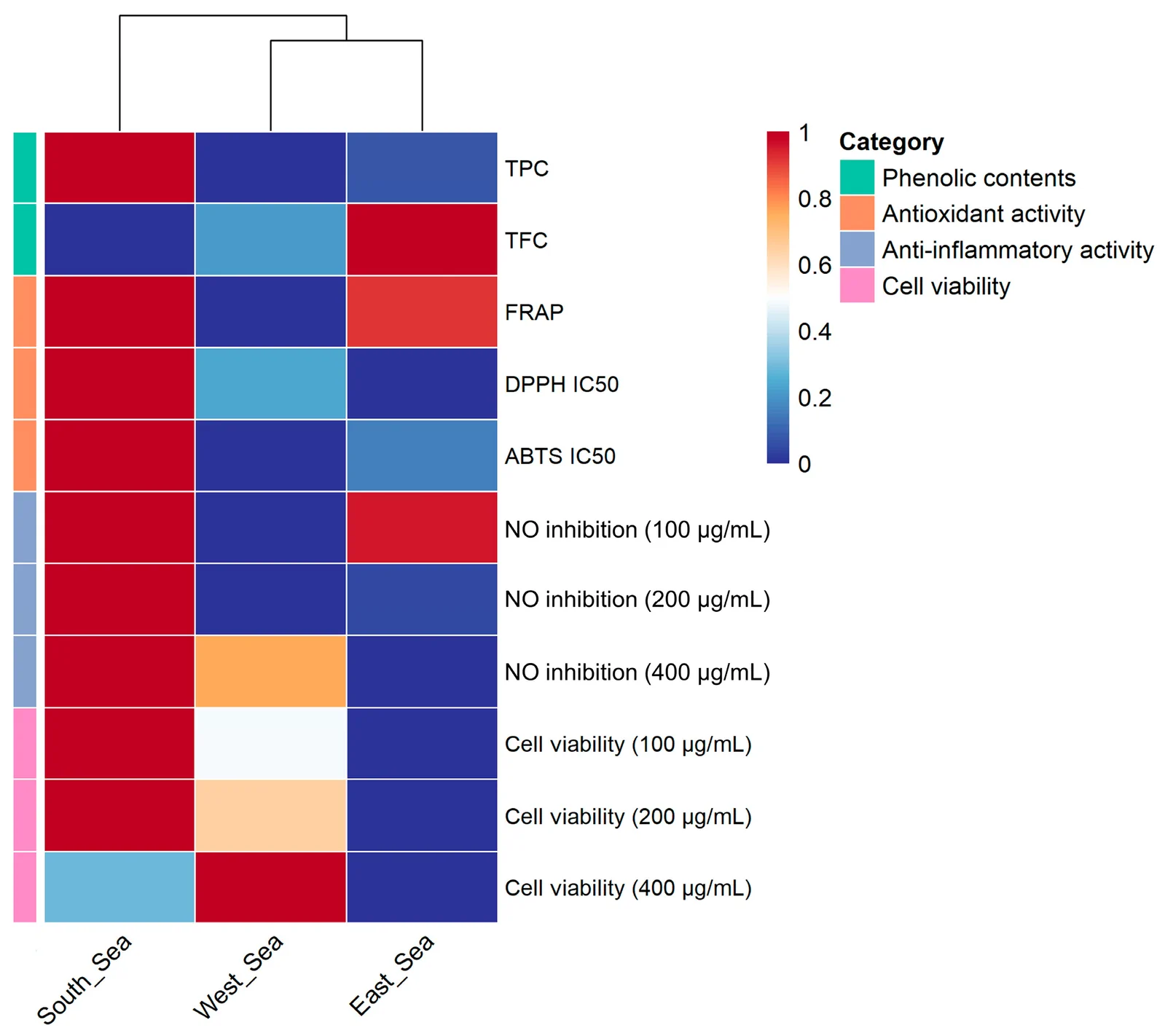
Integrated heatmap and hierarchical clustering of the phenolic contents, antioxidant activities, anti-inflammatory activities, and cell viability of *S. horneri* extracts from different Korean coastal regions. The heatmap was generated using normalized values for TPC, TFC, FRAP, DPPH and ABTS radical-scavenging activities (IC_50_), NO inhibition at 100, 200, and 400 μg/mL, and cell viability at 100, 200, and 400 μg/mL. DPPH and ABTS IC_50_ values were inversely transformed before normalization so that higher normalized scores represent stronger antioxidant activity. NO data were expressed as inhibition relative to the LPS-stimulated control. Values were normalized to a scale of 0–1, with higher scores generally indicating greater activity. Hierarchical clustering of the regional extracts was performed using Euclidean distance and Ward’s minimum variance method (Ward.D2). The color scale represents normalized values from low (blue) to high (red).

**Figure 5:**
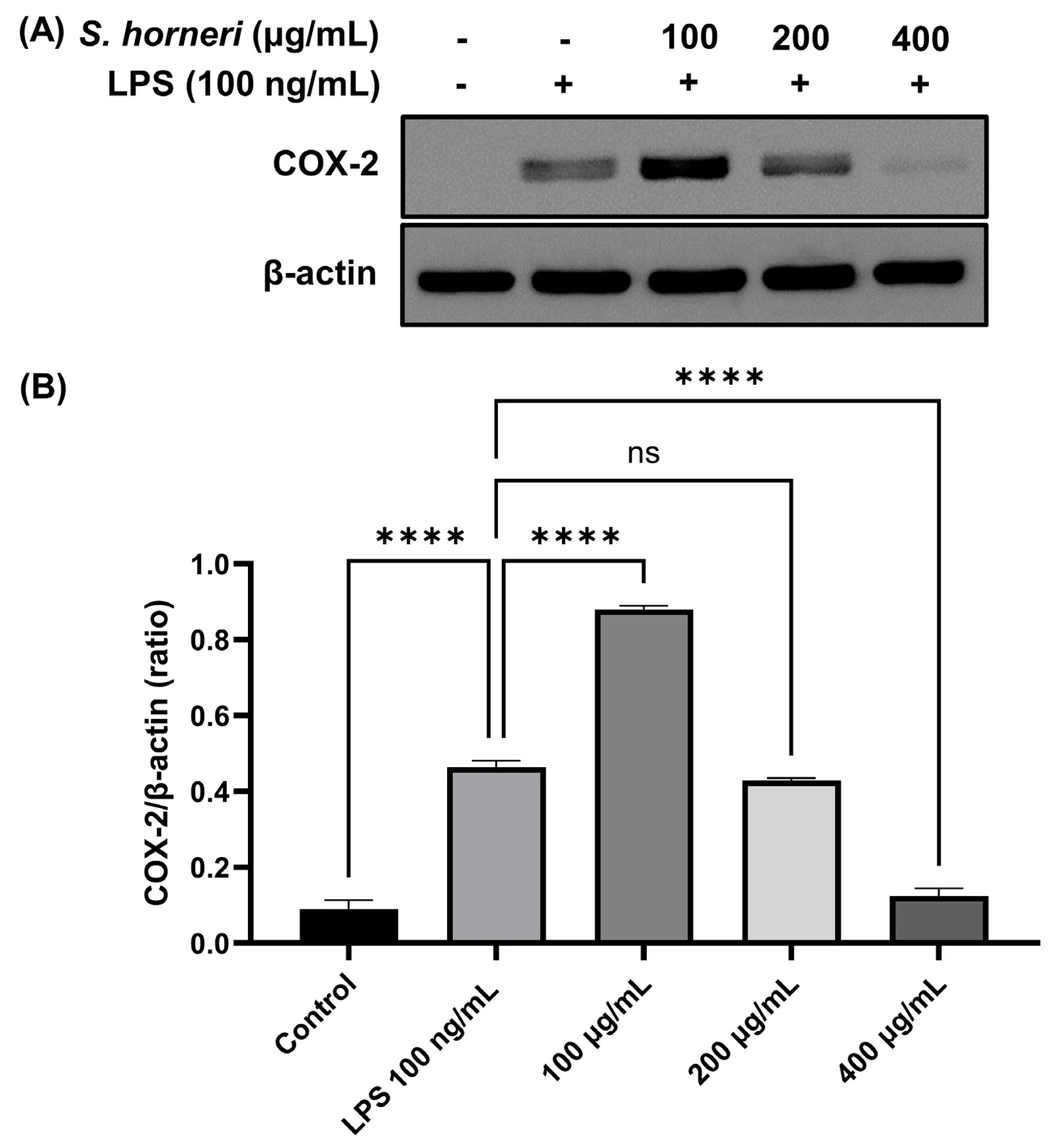
Effect of the South Sea *S. horneri* extract on COX-2 expression in LPS-stimulated RAW 264.7 macrophages. RAW 264.7 cells were treated with the South Sea *S. horneri* extract (100, 200, or 400 μg/mL) and stimulated with LPS (100 ng/mL). (**A**) Protein expression of COX-2 was determined by Western blot analysis, with β-actin used as the loading control. (**B**) Relative COX-2 protein levels were quantified by densitometric analysis and normalized to β-actin. “+” and “−” indicate the presence and absence of the indicated treatment, respectively. Data are presented as the mean ± SD of triplicate measurements. Statistical significance is indicated as **** *p* < 0.0001; ns, not significant.

**Figure 6:**
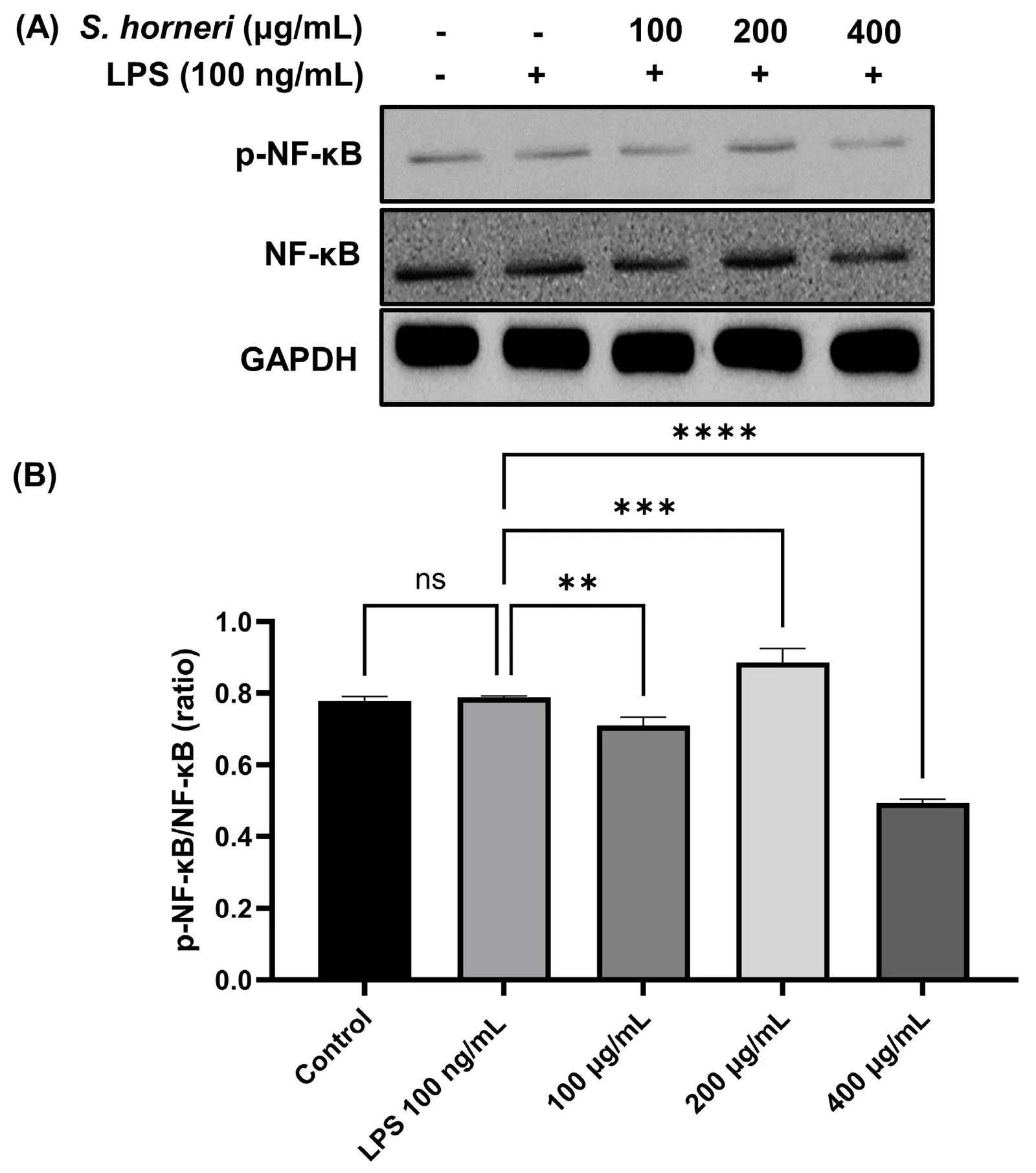
Effect of the South Sea *S. horneri* extract on NF-κB phosphorylation in lipopolysaccharide-stimulated RAW 264.7 macrophages. RAW 264.7 cells were treated with the South Sea *S. horneri* extract (100, 200, or 400 μg/mL) and stimulated with LPS (100 ng/mL). (**A**) p-NF-κB and total NF-κB protein levels in whole-cell lysates were determined by Western blot analysis, with GAPDH used as the loading control. (**B**) NF-κB phosphorylation was quantified by densitometric analysis and expressed as the p-NF-κB/NF-κB ratio. “+” and “−” indicate the presence and absence of the indicated treatment, respectively. Data are presented as the mean ± SD of triplicate measurements. Statistical significance is indicated as ** *p* < 0.01, *** *p* < 0.001, and **** *p* < 0.0001; ns, not significant.

**Figure 7:**
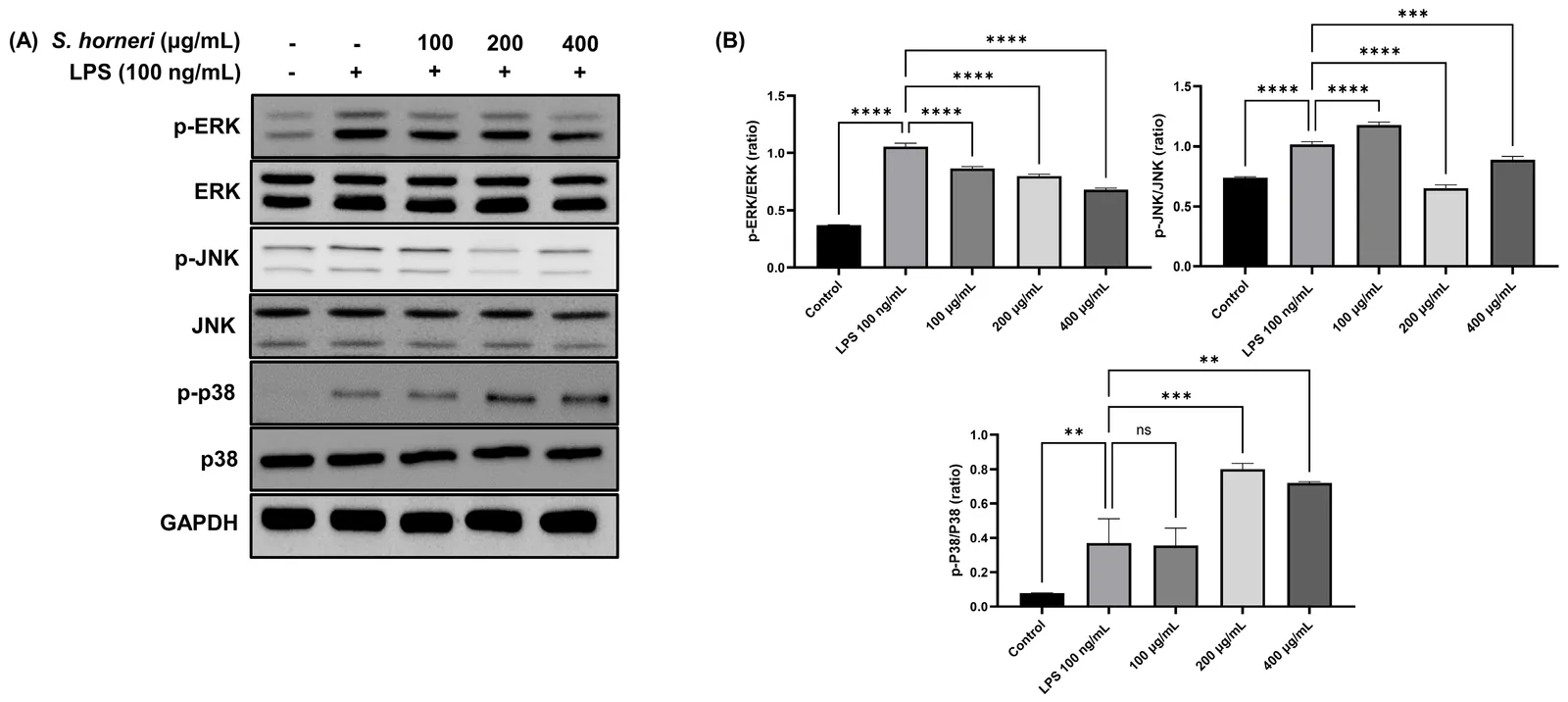
Effect of the South Sea *S. horneri* extract on MAPK phosphorylation in LPS-stimulated RAW 264.7 macrophages. RAW 264.7 cells were treated with the South Sea *S. horneri* extract (100, 200, or 400 μg/mL) and stimulated with LPS (100 ng/mL). (**A**) Protein levels of phosphorylated and total ERK, JNK, and p38 MAPK in whole-cell lysates were determined by Western blot analysis. (**B**) The phosphorylation levels of ERK, JNK, and p38 were quantified by densitometric analysis and expressed as the ratios of phosphorylated to total protein. “+” and “−” indicate the presence and absence of the indicated treatment, respectively. Data are presented as the mean ± SD of triplicate measurements. Statistical significance is indicated as ** *p* < 0.01, *** *p* < 0.001, and **** *p* < 0.0001; ns, not significant.

**Figure 8:**
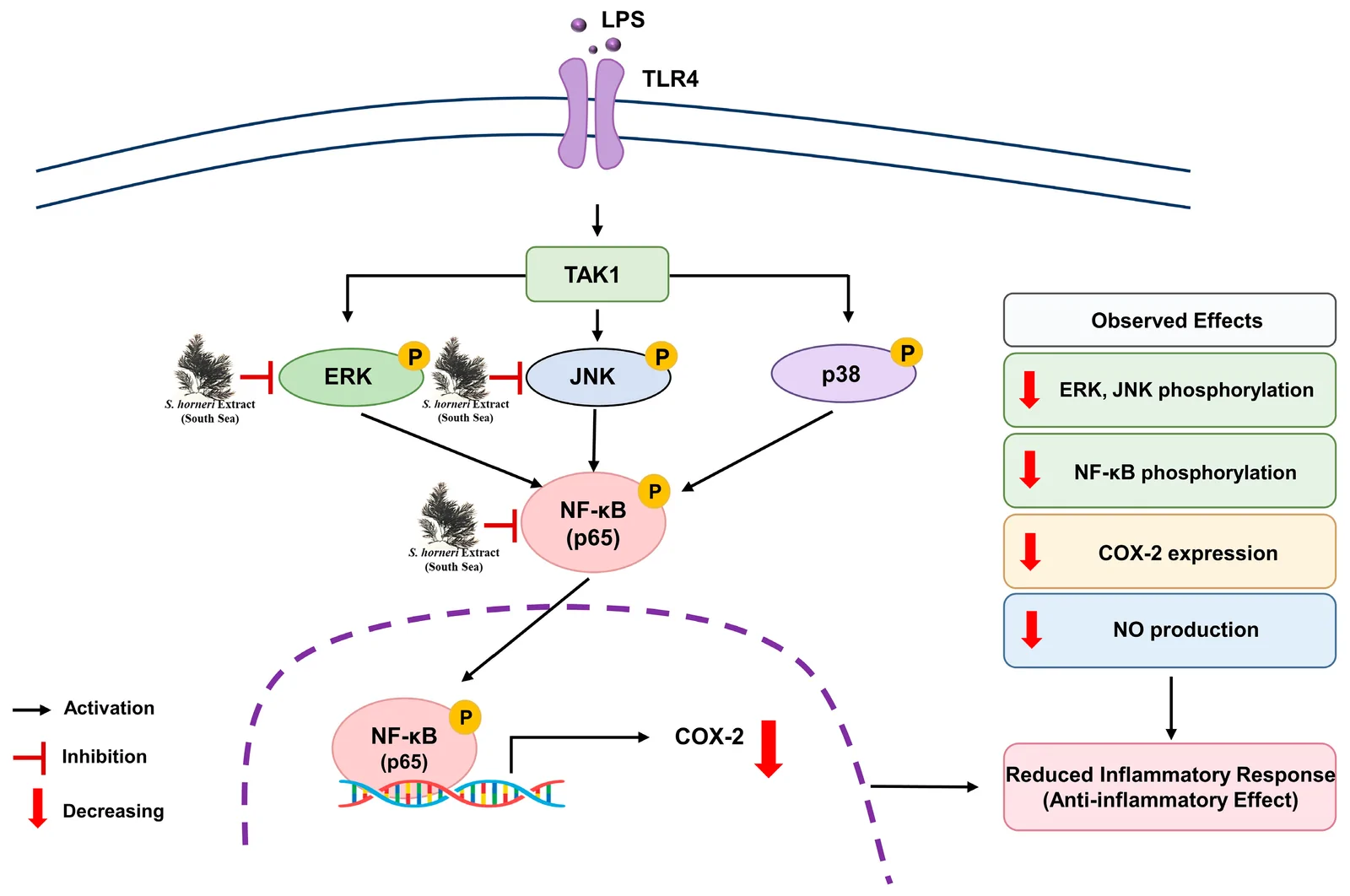
Proposed mechanism underlying the anti-inflammatory effects of the South Sea *S. horneri* extract. The extract may exert anti-inflammatory effects by modulating MAPK/NF-κB signaling, thereby reducing COX-2 expression and NO production in LPS-stimulated RAW 264.7 macrophages. “P” indicates phosphorylation, and different colors and shapes represent the indicated signaling molecules and proteins.

**Table 1:** Information on *S. horneri* extracts obtained from different Korean coastal regions.

Coastal Region	Collection Site	MABIK No.	Collection Data	Part Used	Extraction Solvent	Extraction Method
West Sea	Taean-gun, Chungcheongnam-do	NP30220010	10 June 2021	Whole	70% EtOH	Ultrasonic extraction
South Sea	Jeju-si, Jeju-do	NP30220007	18 April 2022	Whole	70% EtOH	Ultrasonic extraction
East Sea	Yangyang-gun, Gangwon-do	NP30230180	19 April 2023	Whole	70% EtOH	Ultrasonic extraction

## Data Availability

All data generated or analyzed during this study are included in this article.

## Abbreviations

The following abbreviations are used in this manuscript:

ABTS	2,2′-Azino-bis(3-ethylbenzothiazoline-6-sulfonic acid)
COX-2	Cyclooxygenase-2
DMEM	Dulbecco’s modified Eagle’s medium
DPPH	2,2-Diphenyl-1-picrylhydrazyl
ERK	Extracellular signal-regulated kinase
FBS	Fetal bovine serum
FRAP	Ferric reducing antioxidant power
GAE	Gallic acid equivalents
GAPDH	Glyceraldehyde-3-phosphate dehydrogenase
HRP	Horseradish peroxidase
IC_50_	Half-maximal inhibitory concentration
JNK	c-Jun N-terminal kinase
LPS	Lipopolysaccharide
MAPK	Mitogen-activated protein kinase
MTT	3-(4,5-Dimethylthiazol-2-yl)-2,5-diphenyltetrazolium bromide
NF-κB	Nuclear factor-kappa B
NO	Nitric oxide
PBS	Phosphate-buffered saline
QE	Quercetin equivalents
TFC	Total flavonoid content
TPC	Total phenolic content
TPTZ	2,4,6-Tripyridyl-s-triazine
